# Reduction of MicroRNA-206 Contributes to the Development of Bronchopulmonary Dysplasia through Up-Regulation of Fibronectin 1

**DOI:** 10.1371/journal.pone.0074750

**Published:** 2013-09-10

**Authors:** Xiaoying Zhang, Jing Xu, Junjie Wang, Ludwig Gortner, Sheng Zhang, Xiujuan Wei, Jie Song, Yupei Zhang, Qiuping Li, Zhichun Feng

**Affiliations:** 1 Department of Pediatrics, BaYi Children’s Hospital of The General Military Hospital of Beijing PLA, Beijing, P. R. China; 2 Department of Pediatrics, Maternal and Child Health Hospital of Guangxi Zhuang Autonomous Region, Nanning, P. R. China; 3 Burn Center, Changhai Hospital, Second Military Medical University, Shanghai, P. R. China; 4 Pediatric University Hospital, University of Saarland, Homburg, Germany; University of Kansas Medical Center, United States of America

## Abstract

**Objective:**

To characterize microRNA-206 (miR-206) in the development of bronchopulmonary dysplasia (BPD).

**Design/Methods:**

We assessed the expression of miR-206 in BPD mouse lung tissues and blood samples of BPD patients by quantitative real-time PCR. Then, the role of miR-206 in regulating cell biology were examined by XTT assay, flow cytometry, transwell invasion assay, wound healing assay and adhesion assay in vitro. Furthermore, luciferase reporter assay, real-time PCR, western blot and Immunofluorescence staining were performed to figure out the target gene of miR-206.

**Results:**

A reduction in expression of miR-206 was observed in BPD mice compared with controls and in BPD patients compared with controls. miR-206 overexpression significantly induced cell apoptosis, reduced cell proliferation, migration and adhesion abilities, whereas the inhibition of miR-206 expression had the opposite effect. Fibronectin 1 (FN1) is a direct target of miR-206, and fn 1 can be transcriptionally and translationally regulated by miR-206. Down-regulation of miR-206 modulates biological functions of the cells, at least in part, by increasing the level of fn 1. Furthermore, fn 1 expression levels were increased in the BPD mice and BPD patients.

**Conclusions:**

The expression of miR-206 and its target gene, fn 1, may contribute to the progression of BPD.

## Introduction

Babies born prematurely or who experience respiratory problems shortly after birth are at risk for bronchopulmonary dysplasia (BPD), which is a common chronic lung disease with a multifactorial etiology that manifests in preterm neonates. Up to 70% of babies born before 26 weeks of gestation develop BPD [1,2]. Histologically, BPD is characterized by poor alveolarization, abnormal elastin deposition, fibrosis, mesenchymal cell hyperplasia and abnormal capillary growth [1]. Although several lines of evidence indicate that a severely perturbed extracellular matrix (ECM) metabolism contributes to this disorder [3], the underlying pathogenesis is not fully understood, and no evidence-based strategies to prevent or treat BPD are currently available.

MicroRNAs (miRNAs) are 21^~^25 nt long non-coding RNAs that are involved in various biological processes, including cell proliferation, cell death, stress resistance, and tumorigenesis [4]. Using newborn mouse models, we previously demonstrated that miRNAs are associated with lung development and that altered miRNA levels contribute to the development of BPD [5,6]. The mechanism by which functional miRNA modulates the pathogenesis of BPD is not well understood. We report here that miR-206 is down-regulated in BPD patients and BPD newborn mice, and miR-206 targets fibronectin 1 (FN1), an ECM glycoprotein that is involved in cell adhesion and migration processes including embryogenesis, wound healing, metastasis, and host defense [7]. Our results may reflect an important role for miR-206-mediated ECM remodeling during the development of BPD.

## Materials and Methods

### Mice Model

All experiments involving animals were reviewed and approved by the hospital of Beijing Institutional Animal Care and Use Committee (IACUC) and conformed to the guidelines of the National Institutes of Health concerning the care and use of laboratory animals. The experimental BPD mouse model was induced as described elsewhere [5,8]. Animals were euthanized with intraperitoneal sodium pentobarbital after exposure on P2, P7, and P21.

### Subjects and Sample Collection

Twenty patients with BPD, according to the National Institute of Child Health and Human Development (NICHD) guidelines [9] and ten non-BPD age-matched controls were enrolled from clinics at the General Military Hospital of Beijing PLA (Table S1). The study was approved by the Ethics Committee of the General Military Hospital of Beijing PLA. Human blood samples were obtained from these patients and written informed consent was obtained from the guardians of the patients.

### Cells

A549 (Human lung adenocarcinoma epithelial cell line, metastatic cells, purchased from Institute of Basic Medical Sciences Chinese Academy of Medical Sciences) and H441 (Human lung adenocarcinoma epithelial cell line, non-metastatic cells, purchased from Shanghai Xiangf Biotechnology) cells were cultured in 1640 (Gibco-BRL, NY, USA), with 10% fetal bovine plasma (Gibco-BRL, NY, USA). Cells were maintained in a humidified 37°C incubator with an atmosphere of 5% CO_2_.

### Isolation of Lung RNA

Total RNA was isolated from individualwhole lungs (newborn mice on P2, P7, and P21), patient plasma samples (BPD children and controls) and cells (A549 and H441) using miRVana kits (Ambion, Austin, USA) according to the manufacturer’s instructions.

### Reverse Transcription Reaction and Quantitative Real-time PCR

Total RNAs were purified with the Absolutely RNA Nanoprep kit (Stratagene, Amsterdam, The Netherlands). Reverse transcription (RT) reactions and real-time PCR were carried out as we previously described [4]. The relative expression of miRNA compared to *u6* was calculated with the 2^-ΔΔCt^ method. Primers are listed in Table S2. Real-time PCR for FN *1* has been described. Primers are listed in Table S2.

### Construction of pMIR-FN1

pMIR-FN1 (firefly luciferase reporter vector): The 3'-untranslated region (3’-UTR) of the fn*1* gene containing the putative miR-206 target site was cloned into the SacⅠ and Hind Ⅲsite of the pMIR-REPORT™ Luciferase vector (Ambion, USA). All of the oligonucleotide sequences were as shown in Table S2.

### Small Interfering RNA Synthesis

Three small interferingRNA (siRNA, *adr 1-3*) sequences were designed to target regions of FN *1* (sequences shown in Table S2) and synthesized by GenePharma.

### Transient Transfection

Transfections were performed using a Lipofectamine^TM^ 2000 kit (Invitrogen, CA, USA) according to the manufacturer’s instructions and our previous report [4]. Cells (1–3×10^6^ per well) grown to a confluency of 50% to 60% in 10-cm petri dishes were transfected with different double-stranded miRNA mimics, siRNA sequences (600 pmol, GenePharma, Shanghai, China) or their relative mock sequences (listed in Table S2), and the cells were harvested at 24 h, 48 h and 72 h post-transfection.

### Western Blot Analysis

Total soluble proteins (100 µg) extracted from the samples were resolved on 12% (β-actin) or 8% (FN 1) sodium dodecyl sulfate–polyacrylamide gels and transferred electrophoretically to a polyvinylidene fluoride membrane. Blots were blocked with 5% skim milk, followed by incubation with antibodies specific for either FN 1 (1:200, Proteintech, #15613-1-AP) or β-actin (1:200, Cell Signaling, #13E5). Blots were then incubated with goat anti-rabbit secondary antibody (1:5000, Bioworld Technology, #BS13278) and visualized using enhanced chemiluminescence.

### Immunofluorescence

Cells or mouse lung tissue samples were incubated with a rabbit polyclonal anti-FN 1 antibody (Proteintech, #15613-1-AP) at a dilution of 1:50 as the primary antibody. A goat anti-rabbit IgG conjugated with FITC (Jackson ImmunoResearch, #88370, detection for cells) or TRITC (Jackson ImmunoResearch, #85851, detection for lung tissues) was used as the secondary antibody at a dilution of 1:200. Samples were counterstained with Hoechst 33258 and photographed using a confocal microscope (Nikon, C1 Si, Japan).

### Measurement of Cell Proliferation

Cells transfected with miRNAs or siRNAs (1×10^4^ per well) were plated in 96-well plates, and the cell proliferation was measured with a proliferation kit (XTT II, Boehringer Mannheim, Germany) [4]. Optical density was read with a microplate reader (BIO-RAD, USA).

### Measurement of Cell Apoptosis by Flow Cytometry

Cells transfected with miRNAs or siRNAs (1×10^5^ per well) were plated in six-well plates. Apoptosis Inducer, Cisplatin (10 mg/ml, Beyotime, Shanghai, China) were added to the culture. After a 24-h incubation apoptosis was measured by ApopNexin^TM^ fluorescein isothiocyanate (FITC) Apoptosis Detection Kit (APT750, Millipore, Temecula, CA) as previously described. Fluorescence due to FITC and PI staining was measured in a flow cytometer (Cytomics FC 500, Beckman Coulter, Brea, CA).

### Cell Migration Assay

Transfected cells were seeded in the medium without plasma in the upper chamber of a Transwell Permeable Supports chamber (Corning, NY, USA) with an 8.0 µm mesh separating the upper and lower chambers. Cells were allowed to migrate into the lower chamber for 6 h at 37°C, and the invasive activity was analyzed as previously described [4,8].

### Wound Healing Assay

Transfected cells (10^6^ cells/ml) were seeded into a plastic 24-well cell culture plate, and when the cells grew to a monolayer, the monolayer was scraped with a 200 µL pipette tip to create an artificial wound field. The surface area of the wound was recorded over time with light microscopy (Nikon, TE2000-S, Japan). Photographs were taken to assess the level of migration in each group of transfected cells. Migration was quantified by counting the total number of cells that migrated toward the original wound field.

### Adhesion Assay

A 96-well-plate was precoated with 5 µg Matrigel^TM^ Matrix (BD Biosciences, MA, USA) before seeding transfected cells. After incubation for 1 hour, the wells were washed twice with PBS to remove nonadherent cells, fixed in paraformaldehyde, and stained with Giemsa stain. Adherent cells were subsequently observed under a microscope, and the number of adherent cells per field was calculated in eight random fields [10].

### Luciferase Reporter Assay

H441 cells (5×10^4^ per well) were seeded in a 24-well plate the day before transfection and transfected with pMIR-FN 1 (500 ng), the Renilla luciferase control vector (50 ng, pRL-TK-Promega), and miR-206 or miR-3965, or mock (15 pmol miRNA mimics, GenePharma). Assays were performed 48 h after transfection using the dual luciferase reporter assay system (Promega, Madison, USA) as we previously described [4].

### Statistical Analysis

Data are presented as the means ± the standard deviation. Student’s t test was used to compare the two groups. The difference was considered statistically signiﬁcant at p<0.05.

## Results

### miR-206 Is Significantly Down-regulated in the BPD Samples

**Figure 1:**
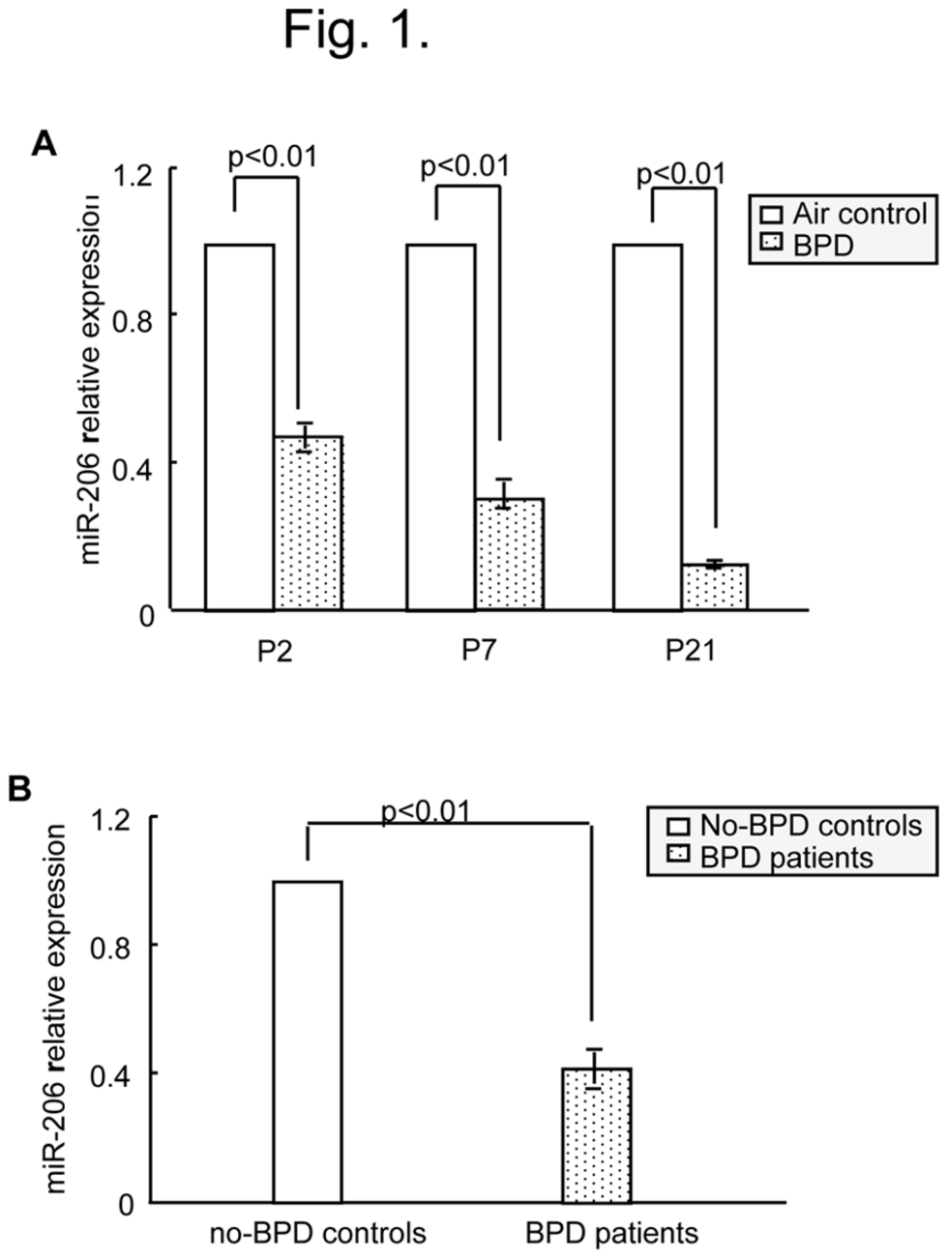
miR-206 had lower expression in BPD. (A) Relative expression of miR-206 for neonatal mice was detected using real-time PCR. The relative expression of miR-206 in BPD mice compared with air-exposed control mice. (B) Relative expression in patient samples of miR-206 in BPD. The specificity of every real-time PCR was assessed with a melting curve.

First, we assessed the expression of miR-206 in mouse lungs on P2, P7, and P21 and found that the lungs in BPD mice exhibited significantly reduced miR-206 expression levels in comparison to air-exposured controls (Figure 1A). On P2, miR-206 decreased by ^~^50% in BPD lungs compared to the lungs of the controls, and on P7 and P21, the expression levels of miR-206 were significantly down-regulated by 67% and 93%, respectively (Figure 1A). Next, we determined whether miR-206 was expressed differentially in human BPD. Blood samples were collected from clinical patients: twenty BPD patients and ten non-BPD matched controls (Table S1). Compared with the non-BPD plasma samples, miR-206 concentrations were significantly lower among the BPD samples (Figure 1B). These data indicated that miR-206 might play a key role in the development of BPD.

### miR-206 Affects Cell Biology In Vitro

**Figure 2:**
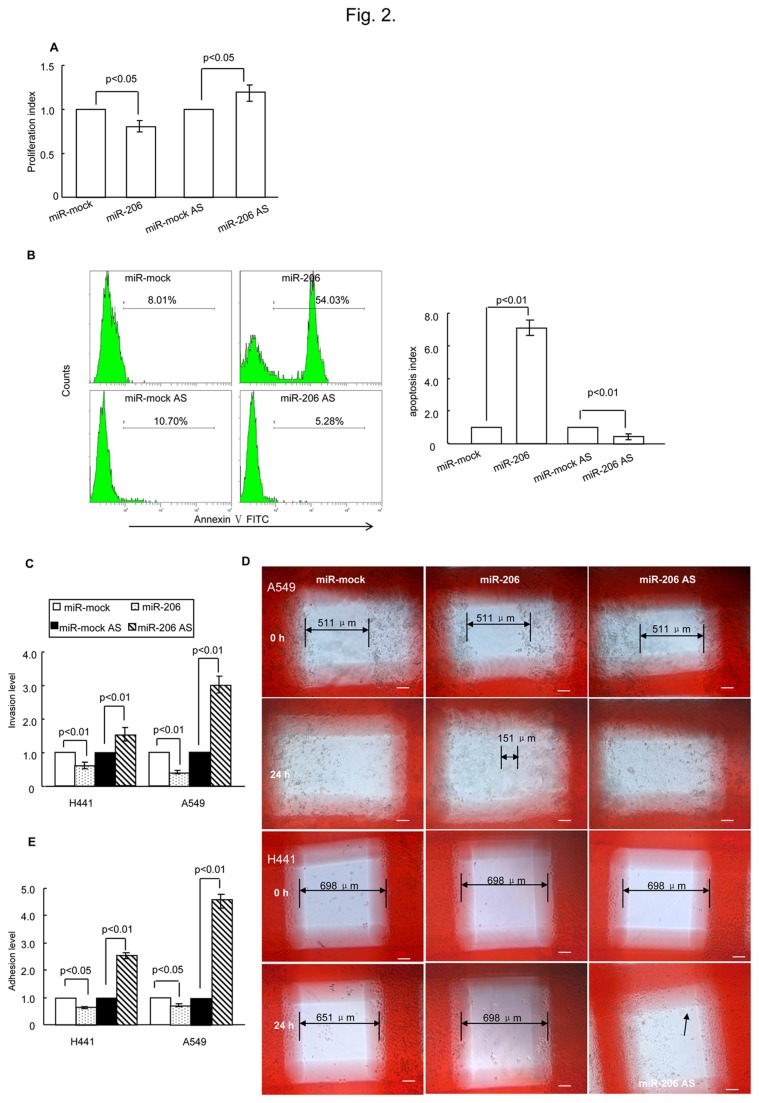
miR-206 affected cell biological functions. (A) miR-206 had a significant effect on cell proliferation as analyzed using XTT (2-3-bis(2-methoxy-4-nitro-5-sulfophenyl)-5-[(phenylamino) carbonyl]-2H-tetrazolium hydroxide) 24 h after transfection. (B) miR-206 was involved in cell apoptosis as assessed by flow cytometry. H441 cells were treated with miRNA and apoptosis inducers was described in Methods. Twenty-four hours later, the cells were collected and analyzed. Profiles on the left are representative of at least three independent experiments. Statistical analysis is shown on the right. (C) Determination of miR-206 involvement in cell invasion by the transwell invasion assay. The relative number of miR-206-trasfected cells (A549 and H441) that migrated compared with miR-mock-transfected cells, and the relative number of miR-206 AS-trasfected cells that migrated compared with mock AS-trasfected cells after 24 h post-transfection. (D) The wound healing assay showed that after 24 h in culture, in A549 cells, the mock group and the miR-206 AS group had covered the artificial wound field, whereas the miR-206 group still had a blank area (double arrowed, length: 151 µm). As for H441 cells, the miR-206 AS group covered the artificial wound field (arrowed), and the mock group and the miR-206 group left the original wound field open (double arrowed, mock length: 651µm, miR-206 length: 698 µm). White bars represent 100 µm. (E) Cell-cell adhesion abilities were determined by an adhesion assay. The relative number of adhesion cells (A549 and H441) after miR-206 transfection compared with cells transfected with miR-mock, and the relative number of adherent cells after miR-206 AS transfection compared with mock AS-transfected cells after 24 h post-transfection. AS, antisense.

Next, we wanted to determine whether miR-206 de-regulation would affect cell biology. The proliferation potential and apoptosis levels of H441 cells transfected with miR-206, miR-206-AS (miR-206 antisense, an inhibitor of miR-206) or their relative mock sequences were analyzed. miR-206 overexpression significantly reduced cell proliferation, and down-regulation of miR-206 significantly increased cell proliferation in vitro (Figure 2A). Furthermore, miR-206 overexpression can induce apoptosis, whereas the inhibition of miR-206 expression had the opposite effect (Figure 2B). Therefore, we evaluated whether miR-206 contributed to the cell migratory potential. Compared with the mock group, cell migration was significantly increased in cells transfected with miR-206-AS, whereas cell migration was significantly suppressed in cells transfected with the miR-206 mimics (Figure 2C). Interestingly, the wound healing assay and the adhesion assay revealed that miR-206 overexpression can greatly reduce cell migratory ability and cell-cell adhesion, especially in metastatic cells (A549) (Figure 2D, E). Thus, these results indicate that down-regulation of miR-206 can promote cell adhesion and migration while also decreasing apoptosis levels.

### miR-206 Represses FN1 Expression

**Figure 3:**
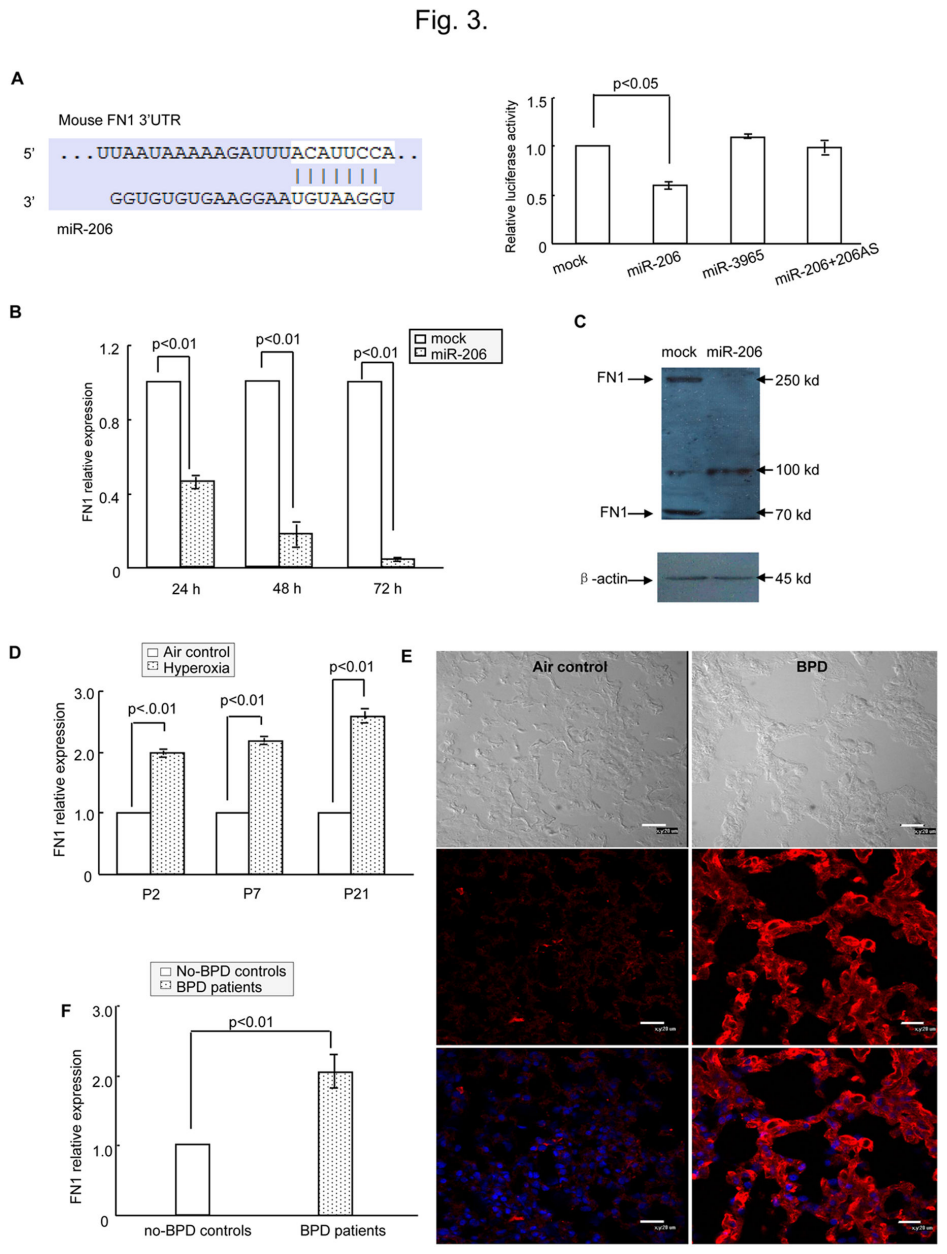
FN 1 was a direct target of miR-206. (A) Left panel shows predicted duplex combination between human FN 1 3’-UTR and miR-206 (upper, sequence of mouse FN 1 3’-UTR including miR-206 binding site; lower, sequence of miR-206). Right panel shows dual luciferase assay of H441 cells cotransfected with pMIR-FN 1 and miRNA (mock, miR-3965, miR-206 or miR-206 + miR-206AS). (B) Real-time PCR of H441 cells transfected with miR-206 or mock to detect FN 1 expression. (C) Western blotting of H441 cells transfected with miR-206 or mock to detect FN 1 expression. (D) Relative expression of FN 1 for neonatal mice was detected using real-time PCR. The relative expression of FN 1 in BPD mice compared with air-exposed control mice. (E) Detection of FN 1 expression in BPD lung and controls by immunohistochemical staining. FN 1 (red) was expressed at higher levels in BPD samples (P21) compared with air-exposed controls (P21). Bars represent 20 µm. (F) Relative expression in patient samples of FN 1 in BPD by using real-time PCR.

To elucidate the mechanisms by which miR-206 affects cell biology, we performed a TargetScan (Release 6.2: June 2012, http://www.targetscan.org/) to help identify miR-206 targets. Among the approximately 800 candidate genes, FN 1 was one of the high-scoring candidates. FN 1 is an ECM component, and the ECM is critical for lung development [7,11]. FN 1 plays a major role in fundamental biological processes such as cell adhesion and migration, maintenance of normal cell morphology, cytoskeletal organization, and cell differentiation [12]. As shown in Figure 3A (left), the FN 1-encoded mRNA contains a 3’-UTR element that is partially complementary to miR-206. To confirm this information, a luciferase reporter assay was performed. miR-206 can effectively inhibit the luciferase activity of pMIR-FN 1, whereas no significant reduction in luciferase reporter activity was observed when cells were co-transfected with miR-3965 (a noncognate miRNA) or mock miRNAs (Figure 3A, right). Finally, we tested FN 1 mRNA and protein levels in H441 cells by real-time PCR (Figure 3B) and western blotting (Figure 3C) with and without miR-206 mimic transfection to determine the mechanism underlying miR-206 action. As shown in Figure 3B and 3C, FN *1* mRNA levels and protein levels decreased substantially after treatment with miR-206. These results strongly indicate that FN 1 is a direct target of miR-206, and FN 1 can be transcriptionally and translationally regulated by miR-206.

As we previously reported, a significant elevation of a number of genes associated with ECM remodeling and fibrosis (TGF-β1, collagen 1α, TIMP-1, IL-1 and TNF-α) was detected in BPD mice versus controls [8]. Further, we found in this study that FN 1 mRNA levels were significantly induced after hyperoxia exposure compared with air-exposed controls (Figure 3D). On P2, P7 and P21, FN 1 increased by ^~^2-fold, 2-fold, and > 2-fold, respectively, in BPD lungs compared to the lungs of air-exposed controls (Figure 3D). The protein levels of FN 1 were likewise much higher in the lung tissues of BPD mice than in controls, and this result was concordant with RNA changes (Figure 3E). Interestingly, FN *1* mRNA was significantly increased in BPD patients relative to controls, and this increase coincided with reduced miR-206 expression (Figure 3F). Taken together, these observations indicate that low levels of miR-206 expression specifically contribute to the pathogenesis of BPD by inducing FN 1 expression in BPD in vivo.

### Down-regulation of FN1 Inhibits the Migration and Invasion Capability of Cells In Vitro

**Figure 4:**
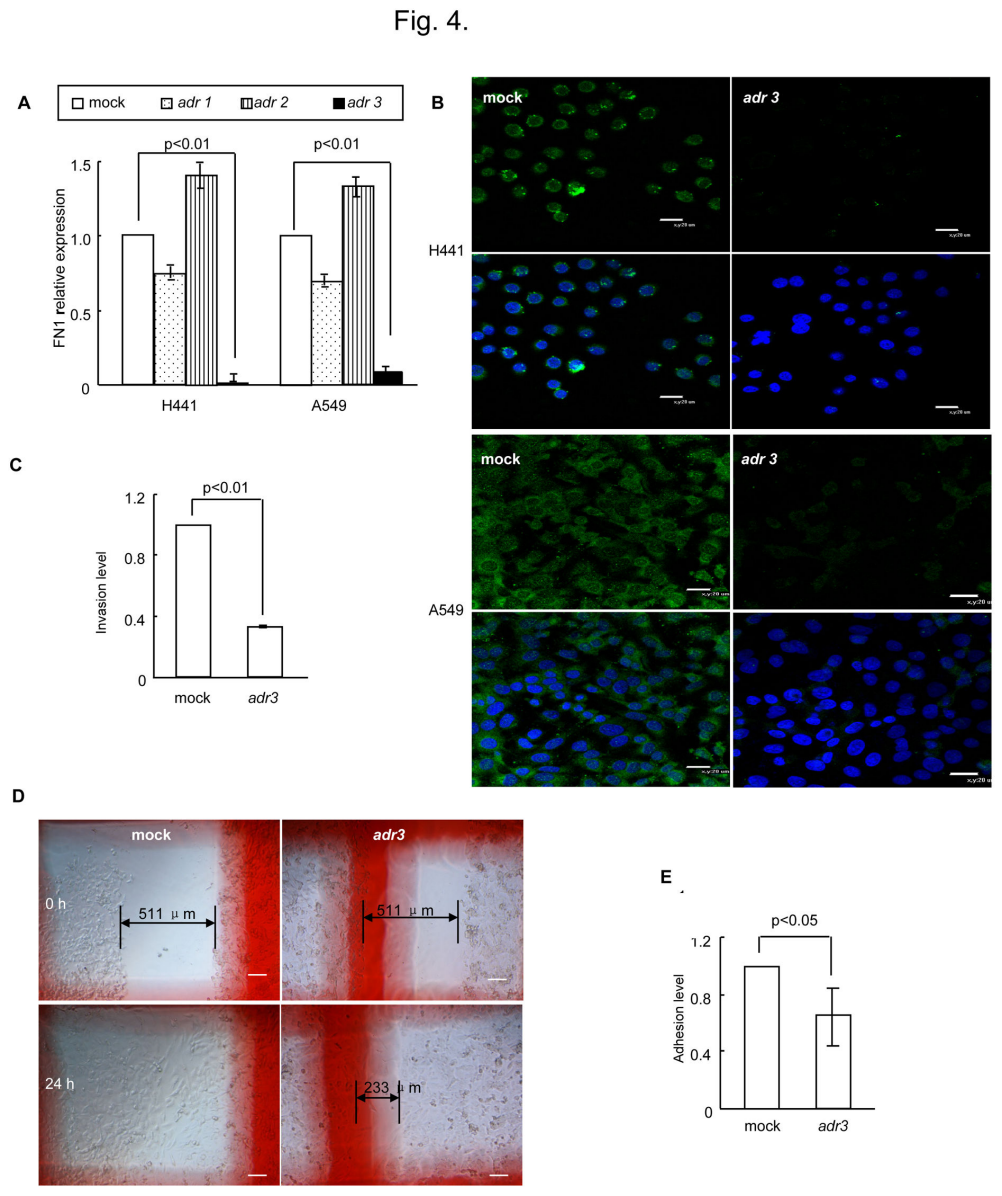
FN 1 regulated cell metastasis In Vitro. (A) Real-time PCR analysis of FN *1* mRNA in cells 24 h after transfection with siRNAs. Compared with mock-transfected H441 and A549 cells, *adr 3* inhibited FN 1 gene expression down by 99% and 90%, respectively. (B) Detection of FN 1 expression in cells by immunohistochemical staining. FN 1 (green) was expressed at high levels in H441 cells, and the fluorescent signal was greatly decreased when the cells transfected with *adr 3*. Bars represent 20 µm. (C) Down-regulation of FN 1 suppressed the invasive properties of A549 cells as visualized by the transwell assay. A549 cells were transfected with *adr 3* or the mock sequence, and after 24 h in culture, the invasion levels of *adr 3* relative to the mock sequence were shown. (D) Wound healing assay in A549 cells showed that after 24 h in culture, the mock group had covered the artificial wound field, whereas the *adr 3* group still left a blank area (double arrowed, length: 233 µm). White bars represent 100 µm. (E) Down-regulation of FN 1 suppressed cell-cell adhesion properties of A549 cells as revealed by the adhesion assay. A549 cells were transfected with *adr 3* or the mock sequence, and after 24 h in culture, the adhesive levels of *adr 3* relative to mock sequence were shown.

After determining that FN 1 was the target of miR-206, we next sought to determine the effect of FN 1 on cell biology in vitro. We first transfected three different siRNA sequences against human FN *1* (*adr* 1-3) into H441 and A549 cells. Twenty-four hours later, the level of FN *1* mRNA in cells was quantified by real-time PCR, and surprisingly, FN 1 gene expression was significantly inhibited by *adr 3* compared with mock-transfected cells (Figure 4A). The reduction in FN 1 protein expression mediated by *adr 3* was further confirmed by immunofluorescence staining (Figure 4B); thus, *adr 3* was used in the following in vitro analyses. The XTT assay and flow cytometry revealed that reduced FN 1 expression had no significant effect on cell proliferation or apoptosis in vitro (data not shown). Subsequently, a Matrigel invasion assay and wound healing assay were performed as described above to assess the effect on the migration and invasion capabilities of A549 cells. In comparison with the *adr-mock* (mock) sequence, the silencing of FN *1* by *adr* 3 decreased the invasive properties of these cells (Figure 4C, D). Furthermore, the adhesion assay also revealed that inhibition of FN *1* by *adr* 3 decreased cell adhesion ability (Figure 4E). Together, these data indicated that FN 1 can affect cell migration and adhension significantly, but not cell proliferation and apoptosis.

### Reduction of miR-206 Enhances Cell Invasion Capability through Up-Regulation of FN1

**Figure 5:**
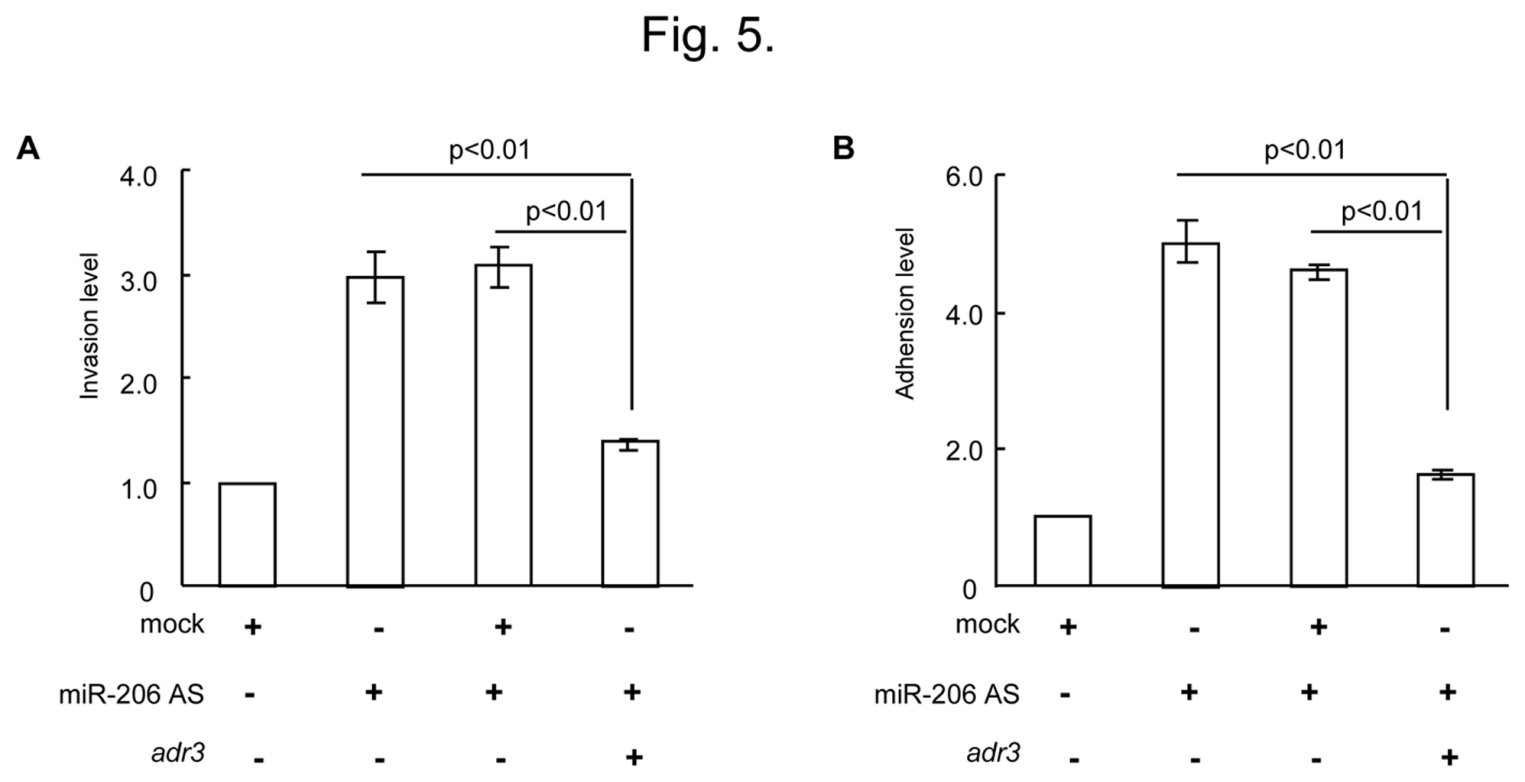
Down-regulated miR-206 involved in the enhanced invasion capability is elicited by FN 1. (A) Determination of miR-206 and *adr 3* involvement in A549 cells invasion by the transwell invasion assay. (B) Determination of miR-206 and *adr 3* involvement in A549 cells adhesion by the adhesion assay. AS, antisense.

To demonstrate whether miR-206 is involved in the reduced invasion capability elicited by FN 1, A549 cells were then either transfected with miR-206-AS or cotransfected with *adr 3* against FN *1* (miR-206-AS + *adr3*) or the *adr*-mock sequence (miR-206-AS + mock). As shown in Figure 5A, transfection of miR-206-AS dramatically enhanced invasive activity compared to the mock group (increased by > 3-fold), and this result was consistent with data illustrated in Figure 2C. Remarkably, the invasion of miR-206-inhibiting cells was markedly inhibited by the transfection of *adr 3*, whereas migration was not altered significantly in cells that were cotransfected with the *adr*-mock sequence. In addition, the adhesion ability induced by miR-206-AS can also be attenuated by *adr 3* (Figure 5B). This result strongly suggests that down-regulation of miR-206 modulates biological functions of the cells, at least in part, by increasing the level of FN 1.

## Discussion

We previously characterized the profile of miRNAs in the lungs of newborn mice with BPD and determined the miRNA profile to be different than controls. Among these miRNAs, we found in this study that BPD mice had decreased expression of miR-206 compared to the air-exposed control mice. Furthermore, the decreased expression of miR-206 was also reflected by low concentrations in the plasma of BPD patients. In addition, the data suggests that FN 1 activity is up-regulated in patients. Together with the in vitro cell data showing a role for miR-206 and FN 1 in modulating cell properties, these data support the hypothesis that altered miR-206 expression and increased FN 1 activity contribute to the development of BPD.

Recent direct evidence suggests that miR-206 is correlated with potential metastatic cancers, such as lung cancer [13], gastric cancer [14], and breast cancer [15,16], through modulating cell proliferation, apoptosis and invasion. Consistently, we observed that when miR-206 was over expressed in H441 cells, cell proliferation, invasion and wound healing levels were notably attenuated, and apoptosis was significantly increased. Furthermore, down-regulated miR-206 had the opposite effect suggesting that miR-206 can significantly affect cellular biological function. Other studies indicate that miR-206 plays an essential role in cell differentiation, especially for muscle differentiation [17–19] and myoblast differentiation [20,21]. Stahlhut C et al. found that miR-206 can regulate angiogenesis by modulating VegfA expression in zebra fish [22]. Moreover, Lewis A et al. reported that miR-1/ -206 was associated with chronic obstructive pulmonary disease (COPD) [23]. A Shh/miR-206/BDNF cascade coordinating innervation and formation of airway smooth muscle has also been demonstrated [24]. In the study described herein, we found that abnormal miR-206 was significantly associated with BPD. Interestingly, we found that miR-206 inhibited the expression of FN 1 through directly interacting with the 3'-UTR of FN 1, and miR-206 mediated cell invasion and wound healing effects at least in part through FN 1, a ubiquitous ECM glycoprotein that is abundant in injured tissues and has many putative roles in wound repair [25].

Previous investigations have found that abnormal remodeling of the ECM has been implicated in the pathogenesis of BPD. Recent data from our laboratory has shown that excessive production and accumulation of collagen appeared around the airways, blood vessels, and in the alveolar regions of BPD mice [8]. In addition, a number of genes associated with ECM remodeling and fibrosis, such as transforming growth factor-β1 (TGF-β1), collagen 1α, and tissue inhibitor of metalloproteinases 1 (TIMP-1), were greatly dysregulated with the development of BPD [8], and these observations were consistent with other reports [3]. In this study, FN 1 expression was increased at the mRNA and protein levels both in the lung tissues of BPD mice and the plasma of BPD patients. As a main ECM component, FN 1 also has angiogenic properties and can directly and indirectly interact with other key regulators of ECM proteins, such as TGF-β1 and VEGF [26,27]. Furthermore, FN 1 is believed to be induced by inflammation [28,29], and numerous studies have linked inflammation with an increase in BPD [30,31]. Therefore, these data support the notion that FN 1 is central to the development of BPD.

We proposed that, as a consequence of the down-regulation of miR-206, the up-regulation of FN 1 observed in a mouse model of BPD and in patients with BPD may play a vital role in ECM remodeling and, thus, contribute to the progression of BPD. Our findings establish a new molecular mechanism with miR-206/FN1 regulating the pathogenesis of BPD that may be helpful in developing an effective treatment against BPD.

## Supporting Information

Table S1 **Clinical characteristics of patients with and without BPD.** H = hours; D = days; wk = weeks; M = male; F= female; ND = not determined; BPD = bronchopulmonary dysplasia; ROP = retinopathy of prematurity; RDS=neonatal respiratory distress syndrome; PDA = patent ductus arteriosus; IVH = intraventricular hemorrhage; HIE = hypoxic-ischemic encephalopathy.
* compared with non-BPD patients.
(DOC)

Table S2 **Oligonucleotide sequences for miR-206 and FN1.** F: forward primer; R: reverseprimer; AS: antisense
(DOC)
