# Supplementary material for: Reduction of MicroRNA-206 Contributes to the Development of Bronchopulmonary Dysplasia through Up-Regulation of Fibronectin 1
Source: PLoS One. 2013 Sep 10;8(9):e74750. doi: 10.1371/journal.pone.0074750 (PMC3769311; doi:10.1371/journal.pone.0074750)
Supplement: Table S1 — Clinical characteristics of patients with and without BPD. H = hours; D = days; wk = weeks; M = male; F= female; ND = not determined; BPD = bronchopulmonary dysplasia; ROP = retinopathy of prematurity; RDS=neonatal respiratory distress syndrome; PDA = patent ductus arteriosus; IVH = intraventricular hemorrhage; HIE = hypoxic-ischemic encephalopathy. * compared with non-BPD patients. (DOC) [file pone.0074750.s001.doc]

Table S1. Clinical characteristics of patients with and without BPD

| Number | M/F | Gestational age, wk | Birth weight, g | Days MV, D | Days FiO2≥0.6,D | Days FiO2<0.6, D | Diagnosis | Outcome |
| --- | --- | --- | --- | --- | --- | --- | --- | --- |
| non-BPD patients | | | | | | | | |
| 1 | M | 30.0 | 1300 | 10 D | 8 D | 10 D | RDS, pneumonia, IVH, PDA | Survived |
| 2 | F | 29.1 | 1800 | 40 D | 1 D | 1 D | ROP | Survived |
| 3 | M | 29.9 | 1540 | 0 | 4 D | 3 D |  | Survived |
| 4 | M | 30.0 | 1350 | 0 | 0 | 2 |  | Survived |
| 5 | M | 28.0 | 1100 | 8 | 4 | 12 | RDS, ROP | Survived |
| 6 | F | 29.0 | 1200 | 14 | 2 | 1 | ROP | Survived |
| 7 | M | 28.0 | 1220 | 17 | 3 | 0 | RDS, pneumonia, IVH, PDA, ROP | Survived |
| 8 | F | 28.0 | 1115 | 0 | 9 | 4 | PDA | Survived |
| 9 | M | 29.0 | 1285 | 13 | 2 | 0 | RDS | Survived |
| 10 | M | 29.0 | 1200 | 17 | 5 | 2 | ROP | Survived |
| Mean ± SE 29.0±0.8 1311±213 | | | | | | | | |
| BPD patients | | | | | | | | |
| 1 | M | 30.3 | 1030 | ND | 37 | 3 | BPD, ROP | Survived |
| 2 | M | 28.0 | 1300 | 26 | 23 | 15 | BPD, RDS, PDA, IVH, HIE | Survived |
| 3 | M | 30.3 | 1500 | 18 | 20 | 7 | BPD | Survived |
| 4 | M | 28.3 | 1150 | 16 | 14 | 31 | BPD, RDS, PDA, IVH, ROP, pulmonary hemorrhage | Survived |
| 5 | M | 29.0 | 925 | 11 | 10 | 35 | BPD, RDS, PDA, gastrointestinal bleeding, septic shock, sepsis, ROP | Survived |
| 6 | M | 28.1 | 1190 | 15 | 17 | 21 | BPD, RDS, ROP, PDA, pulmonary hemorrhage | Survived |
| 7 | F | 31.0 | 1550 | 10 | 13 | 7 | BPD, RDS, PDA | Survived |
| 8 | F | 30.6 | 1100 | 14 | 13 | 5 | BPD, RDS, IVH, anemia | Survived |
| 9 | F | 31.7 | 1590 | 16 | 16 | 2 | BPD, RDS, PDA | Survived |
| 10 | M | 30.4 | 1270 | 12 | 11 | 5 | BPD, RDS, pneumonia, pulmonary hemorrhage, sepsis | Survived |
| 11 | F | 28.0 | 850 | 42 | 41 | 21 | BPD, RDS, pneumonia, PDA, sepsis, ROP | Survived |
| 12 | M | 28.3 | 1000 | 11 | 12 | 39 | BPD, RDS, pneumonia | Survived |
| 13 | F | 26.0 | 1040 | 26 | 18 | 27 | BPD, RDS, PDA, PDA, ROP, IVH, | Survived |
| 14 | M | 29.0 | 1200 | 13 | 13 | 13 | BPD, RDS, PDA, pneumonia | Survived |
| 15 | M | 26.0 | 1140 | 18 | 20 | 38 | BPD, RDS, pneumonia, pulmonary hemorrhage, PDA, IVH, ROP, | Survived |
| 16 | F | 27.6 | 1090 | ND | ND | 26 | BPD, ROP | Survived |
| 17 | F | 28.0 | 950 | 24 | 24 | 16 | BPD, PDA, PDA | Survived |
| 18 | F | 27.1 | 900 | 22 | 19 | 55 | BPD, RDS, pulmonary hemorrhage, PDA, PDA, IVH, ROP | Survived |
| 19 | M | 28.9 | 1200 | 53 | 50 | 20 | BPD, ROP | Survived |
| 20 | M | 27.0 | 960 | 5 | 7 | 25 | BPD, RDS, pneumonia, sepsis, PDA, ROP | Survived |
| Mean ± SE 28.7±1.6 1147±212  **p* value: 0.471 0.056 | | | | | | | | |

H = hours; D = days; wk = weeks; M = male; F= female; ND = not determined; BPD = bronchopulmonary dysplasia; ROP = retinopathy of prematurity; RDS=neonatal respiratory distress syndrome; PDA = patent ductus arteriosus; IVH = intraventricular hemorrhage; HIE = hypoxic-ischemic encephalopathy.

* compared with non-BPD patients.
