# Supplementary material for: Reduction of MicroRNA-206 Contributes to the Development of Bronchopulmonary Dysplasia through Up-Regulation of Fibronectin 1
Source: PLoS One. 2013 Sep 10;8(9):e74750. doi: 10.1371/journal.pone.0074750 (PMC3769311; doi:10.1371/journal.pone.0074750)
Supplement: Table S2 — Oligonucleotide sequences for miR-206 and FN1. F: forward primer; R: reverseprimer; AS: antisense (DOC) [file pone.0074750.s002.doc]

Table S2. Oligonucleotide sequences for miR-206 and FN1.

| Name | Sequence |
| --- | --- |
| miR-206 | Stem-loop: 5’-GTCGTATCCAGTGCGTGTCGTGGAGTCGGCAATTGCA  CTGGATACGACccacac-3’  F: 5’- GGGTGGAATGTAAGGAAGT-3’  R: 5’- TGCGTGTCGTGGAGTC-3’ |
| U6 | F: 5’- GCTTCGGCAGCACATATACTAAAAT-3’  R: 5’- CGCTTCACGAATTTGCGTGTCAT-3’ |
| FN1 | F: 5’- TGA TCA CAT GGA CGC CTGC -3’  R: 5’- GAG TCA AGC CGG ACA CAA CG -3 |
| GAPDH | F: 5’- GACCACTTTGTCAAGCTCATTTCC -3’  R: 5’- GTGAGGGTCTCTCTCTTCCTCTTGT -3’ |
| pMIR-FN1 | F:5’-CAAGACAAGTGTTTTAATAAAAAGATTTACATTCCATGATGTGGA  CGTCATTTCTTTTA-3’  R:5’-AGCTTAAAAGAAATGACGTCCACATCATGGAATGTAAATCTTTTT  ATTAAAACACTTGTCTTGAGCT-3’ |
| miRNA mimics | |
| miR-206 | Sense: 5’-UGGAAUGUAAGGAAGUGUGUGG-3’  Antisense: 5’-ACACACUUCCUUACAUUCCAUU-3’ |
| miR-mock | Sense: 5’-UUCUCCGAACGUGUCACGUTT-3’  Anti-sense: 5’-ACGUGACACGUUCGGAGAATT-3’ |
| miR-206 AS | 5’-CCACACACUUCCUUACAUUCCA-3’ |
| miR-mock AS | 5’- CAGUACUUUUGUGUAGUACAA-3’ |
| siRNA | |
| adr 1 | F: 5’-GUCCUGUCGAAGUAUUUAUTT-3’  R: 5’-AUAAAUACUUCGACAGGACTT-3’ |
| adr 2 | F: 5’-CGGGCAUUGACUAUGAUAUTT-3’  R: 5’-AUAUCAUAGUCAAUGCCCGTT-3’ |
| adr 3 | F: 5’-CGGGAACCGAAUAUACAAUTT-3’  R: 5’-AUUGUAUAUUCGGUUCCCGTT-3’ |
| adr mock | F: 5’-UUC UCC GAA CGU GUC ACG UTT-3’  R: 5’-ACG UGA CAC GUU CGG AGA ATT-3’ |

F: forward primer; R: reverseprimer; AS: antisense
